# Physicochemical properties, droplet size and volatility of dicamba with herbicides and adjuvants on tank-mixture

**DOI:** 10.1038/s41598-020-75996-5

**Published:** 2020-11-02

**Authors:** Pedro Henrique Urach Ferreira, Leonardo Vinicius Thiesen, Gabriela Pelegrini, Maria Fernanda Tavares Ramos, Matheus Moreira Dantas Pinto, Marcelo da Costa Ferreira

**Affiliations:** Departamento de Ciências da Produção Agrícola, UNESP, Jaboticabal Campus, Jaboticabal, SP Brazil

**Keywords:** Leaf development, Environmental monitoring, Chemical physics, Abiotic, Plant sciences, Plant stress responses

## Abstract

The adoption of dicamba-tolerant soybean varieties has increased the concern and demand for new drift and volatility reduction technologies. Potential spray nozzles and adjuvants should be studied to determine its effects on drift and volatility of dicamba tank-mixtures. The objective of this study was to evaluate physicochemical characteristics of spray solutions containing dicamba; to analyze droplet size effect with air induction nozzles; and to assess dicamba volatilization on soybean plants with a proposed methodology. Treatments included dicamba only and mixtures with herbicides and adjuvants. Dicamba mixed with lecithin + methyl soybean oil + ethoxylated alcohol adjuvant had the greatest efficacy potential among treatments considering tank-mixture pH, surface tension, contact angle and droplet size. The MUG11003 nozzle produced the coarsest droplet size and was better suited for drift management among nozzle types. The proposed volatilization methodology successfully indicated dicamba volatilization in exposed soybean plants and among the evaluated treatments, it showed greater volatilization for dicamba with glyphosate + lecithin + propionic acid adjuvant.

## Introduction

Soybean (*Glycine max*) yield is affected by several variables including soil fertility, water availability, temperature, plant population, seed variety, disease, pest management and weed management, among others. Weed control is, however, one of the major concerns to soybean farmers. Potential soybean yield losses by weed interference could reach up to 52% representing near US$ 17 billion considering losses in the US and Canada^1^. Especially when considering broadleaf weed species such as hairy fleabane (*Conyza bonariensis*), pigweeds (*Amaranthus spp.*) and hairy beggarticks (*Bidens pilosa*) which may cause significant yield losses. Reports from several countries indicate that these and other species are resistant to different herbicides, such as glyphosate^2–4^.

To address troublesome broadleaf weeds and weed resistance related problems, studies have been conducted to develop a new soybean variety tolerant to dicamba. In the United States, for example, dicamba-tolerant varieties have been used since 2017 as an auxiliary weed control tool, especially for difficult controlling weeds such as Palmer amaranth (*Amaranthus palmeri*)^5^. The main benefit of adopting dicamba-tolerant varieties is the possibility of diversifying weed management as few alternatives of herbicide-tolerant soybean traits are available. Furthermore, dicamba may control weeds resistant to other modes of action, preventing and delaying new cases of resistance while reducing losses caused by weed competition^6^.

Developed over 50 years ago, dicamba has been extensively used for broadleaf weed control in crops such as corn (*Zea mays*) and sugarcane (*Saccharum officinarum*). However, its use has been restricted due to volatility issues and drift damage to sensitive crops^7^. Currently, new dicamba formulations have significantly reduced herbicide volatility when compared to previous formulations. The dimethylamine (DMA) salt used in initial formulations is more volatile than a diglycolamine (DGA) salt^6^. Now, new formulations composed of N, N-bis-(3-aminopropyl) methylamine (BAPMA) presents lower volatility than older formulations^8^.

Several authors have studied the conditions that intensify herbicide volatility, including tank-mixture, weather conditions during application, spray nozzles, and tank/spray system cleaning. Herbicide mixtures and adjuvants with dicamba may alter tank-mixture pH. Reductions of up to 2 units in the pH were observed when ammonium sulfate was added to the tank-mixture^9^. In another study, losses of dicamba in the environment were evaluated under stable and unstable atmospheric conditions. The addition of glyphosate to the tank-mixture increased losses of dicamba in the environment under both atmospheric scenarios, while detecting dicamba in air for up to 72 h after application^10^. Other researchers have also observed that dicamba and glyphosate mixture increased the presence of dicamba in air by 3 to 9 times, with a decrease occurring only at temperatures below 15 °C^11^. In another case, studying the efficacy of dicamba in relation to the droplet size, researchers found that 395 µm droplet size achieved better weed control than other droplet sizes^12^. However, it was observed that similar sizes increased drift^12^. Droplet sizes close to 620 µm were recommended as weed control was maintained above 90%^12^.

Drift reduction adjuvants and nozzles have been studied to potentially decrease even more dicamba volatility and drift. However, before using any adjuvant, it is necessary to understand how it may benefit the spray solution or improve pest control efficacy. Adjuvants can be classified as evaporation reduction substances, penetrating agents, adhesives, spreaders, anti-foaming agents, drift reducers, and conditioners^13^, besides other function categories. It has been observed, for example, that combinations of dicamba with different adjuvants have the potential to reduce pH and increase droplet size and uniformity in addition to other physicochemical effects^9,14^.

Studying adjuvants tank-mixtures and spray nozzles is necessary as each combination may alter droplet size differently, in special when using air induction nozzles. It has been observed, for instance, that such nozzles do not behave similarly to conventional nozzles with respect to droplet formation^15^. The induction of air for these nozzles varies depending on the properties of the tank-mixture^15^. Thus, studies with tank-mixtures and spray nozzles are extremely important to better understand and predict the behavior of dicamba herbicide in the environment. Such experiments are also necessary to reduce potential losses while increasing dicamba applications efficacy.

The objectives of this study were to characterize dicamba mixtures with glyphosate, saflufenacil and adjuvants; to evaluate the potential of air induction nozzles for drift reduction; and evaluate volatility of dicamba tank-mixtures with a simple proposed methodology.

## Material and methods

The study was divided into two experiments. The first experiment involved laboratory evaluations using combinations of herbicides and adjuvants for the characterization of electrical conductivity factors, pH, surface tension, and contact angle. Moreover, three spray nozzles were used to determine droplet size.

The second experiment included volatility phytotoxicity evaluations in soybean plants. Soybean plants were exposed to dicamba alone and dicamba mixed with other herbicides and adjuvants. The herbicides and adjuvants used are summarized in Table 1 and nozzle types tested are listed in Table 3. Treatment combinations included three herbicides: dicamba (D), potassium glyphosate (R), saflufenacil (H). And included three adjuvants: lecithin + propionic acid (L), lecithin + soybean methylated ester + ethoxylated alcohol (F), and soybean methylated oil (M). Herbicide and adjuvant combination totalized 12 treatments (Table 2).

**Table 1:** Active ingredient, trade name, manufacturer and rate of each herbicide and adjuvant used.

Herbicide active ingredient	Trade name	Product manufacturer	Rate (a.i. ha^−1^)
BAPMA dicamba	not available	not available	540
saflufenacil	Heat	BASF S.A., São Paulo, SP, Brazil	30
potassium glyphosate	Roundup Transorb R	Monsanto, St. Louis, USA	930

Adjuvant active ingredient	Trade name	Product manufacturer	Rate (% v/v)
lecithin + propionic acid	LI700	Fortgreen, Paiçandu, PR, Brazil	0.250
lecithin + soybean methylated ester + ethoxylated alcohol	Fluilflex	Agrichem do Brasil, Ribeirão Preto, SP, Brazil	0.100
soybean methylated oil	MEES	BASF S.A., São Paulo, SP, Brazil	0.333

**Table 2:** Nozzle model, manufacturer and characteristic of each used nozzle.

Common name	Spray nozzle	Nozzle manufacturer	Characteristics
Turbo Teejet Induction	TTI 11003	Teejet Technologies, Wheaton, IL, USA	Air-induction, flat fan, pre orifice
Ultra Lo-Drift Max	ULDM 13003	Pentair-Hypro, New Brighton, MN, USA	Air-induction, flat fan, 130° angle
Magno Ultra Grossa	MUG 11003	Magnojet, Ibaiti, PR, Brazil	Air-induction, flat fan, 30° inclination

### Electrical conductivity and pH

To determine electrical conductivity and pH, spray solutions were prepared and placed in 0.5 L glass beaker for measurement and readings. The electrical conductivity was measured using a Marte MB-11P benchtop conductivity meter (Marte Científica, Santa Rita do Sapucaí, MG, Brazil). The sensor was immersed in spray solution and readings were captured after values stabilized. Measurements of electrical conductivity indicate conductivity of electrical current in a solution (ionic concentration), reflecting the reactivity of the spray solution. pH measurements were conducted using a Quimis Q400RS Bivolt bench pH meter (Quimis, Diadema, SP, Brazil). pH values were used to measure the intensity of liquid acidity and to indicate degradation potential of mixture components by hydrolysis. The experimental design of electrical conductivity and pH evaluations was completely randomized with four repetitions for each treatment.

### Surface tension and contact angle

Static surface tension was determined using a Kruss K20S tensiometer. The equipment was calibrated with deionized water. After calibration, spray solutions were prepared and transferred to a receiving equipment placed in the tensiometer and were adjusted until the tensiometer sensor remained immersed in solution. The equipment platform gradually moved as the sensor separated from the solution surface. Surface tension was obtained in N m^−1^ units after automatic calibration with deionized water.

Contact angle measurements were obtained with the Contact Angle System OCA 15-plus software (DataPhysics Instruments GmbH, Filderstadt, Germany) while automation and processing of computer images were conducted with the SCA20 software (DataPhysics Instruments GmbH, Filderstadt, Germany). Droplets were formed at the needle tip by an automatic triggering injector. Small volumes obtained through a precise syringe plunger movement produced 3 µL volume droplets which were deposited on a paraffin plastic film with a paper surface (Parafilm M, Bemis NA). Droplets were evaluated every second during a total of 60 s. Contact angle results were standardized at 10 s in all treatments for comparison purposes. Static surface tension and contact angle experimental design was complete random with four repetitions for each treatment.

### Droplet size

Three air induction spray nozzles (TTI11003, ULDM13003, and MUG11003) were used for each treatment to determine droplet size. Droplet diameters produced using different tank-mixtures were determined by a Mastersizer S particle size analyzer (Malvern Panalytical Ltd., Malvern, United Kingdom), version 2.19, using laser diffraction method. The optical unit determines droplet size of the sprayed spectrum based on the trajectory deviation suffered by the laser beam when reaching a particle. 250 kPa constant pressure was adjusted and maintained using compressed air. The spray plume crossed the laser beam and was driven transversely by an engine while maintaining a complete sample of the entire sprayed plume. The droplet diameter (µm) where 10, 50 and 90% of the sprayed volume is contained, was measured and represented by values of D_V0.1_, D_V0.5_ and D_V0.9_, respectively. Droplets with high drift potential were analyzed considering the volume percentage of droplets smaller than 150 µm (%V < 150 µm). A coefficient of uniformity or relative span (RS), which represents the distribution of the droplet size spectrum, was analyzed considering the following Eq. (1):

1 $${\text{RS}} = \frac{{\left( {{\text{D}}_{{{\text{V}}0.9}} - {\text{D}}_{{{\text{V}}0.1}} } \right)}}{{{\text{D}}_{{{\text{V}}0.5}} }}$$

The ANSI/ASABE^16^ droplet size standard methodology was adopted to classify droplet spectra of tested nozzles. Reference nozzles were tested with water mixed with surfactant adjuvant (0.1% v/v), TA35 adjuvant (Inquima, Cambé, PR, Brazil). The TTI11003, ULDM13003 and MUG11003 nozzles were tested at constant pressure of 250 kPa.

### Dicamba volatility

A new methodology was proposed to evaluate herbicide volatility based on methodology recommendations by Reynolds^17^ and adaptations described by Ouse et al.^18^. Herbicides and adjuvant tank-mixtures combinations (Table 1) were applied using the MUG 11003 nozzle. This nozzle was selected because it had the highest D_V0.5_ (µm) value, the lowest volume percentage of droplets smaller than 150 µm (%V < 150 µm), and the lowest relative span (RS) among the three nozzle types evaluated (Table 3).

**Table 3:** List of treatments used in the study, composed by twelve tank-mixtures of herbicides and adjuvants.

Herbicides	Adjuvants			
	No adjuvant	LI700	Fluilflex	MEES
Dicamba	D	D + L	D + F	D + M
Dicamba + Roundup Transorb R	D + R	D + R + L	D + R + F	D + R + M
Dicamba + Roundup Transorb R + Heat	D + R + H	D + R + H + L	D + R + H + F	D + R + H + M

The water only (W) treatment is not included. Abbreviations were as follows: W = Water; D = Dicamba; L = LI700; F = Fluilflex; M = MEES; R = Roundup Transorb R; H = Heat.

The experiment was performed in duplicate, on October 25th and 28th, 2019, respectively. Plastic trays (0.265 × 0.230 × 0.045 m) were filled with 1.0 kg of red latosol and submitted to surface irrigation of 5.9 mm water film (300 mL per tray). Applications in each treatment were performed 4 h after soil irrigation, with compressed air in a pressurized costal sprayer at 250 kPa, at 2.38 m s^−1^ speed, and application volume of 150 L ha^−1^. Spray system pressure was regulated by compressed air instead of CO_2_ to avoid variations on tank-mixture pH^19^. Temperature and relative humidity were measured at each application treatment and averaged 25.6 °C and 47%, respectively, on October 25, 2019 whereas on October 28, 2019 averaged 26.0 °C and 52%, respectively. The experimental design was entirely random, with four repetitions. Each repetition consisted of two pots with one V4 stage soybean plant in each, and a tray containing soil treated with each tank-mixture. Soybean stages V4 and R2 were considered because of the maximum reductions in plant height and yield of plants exposed to dicamba^20^.

**Figure 1:**
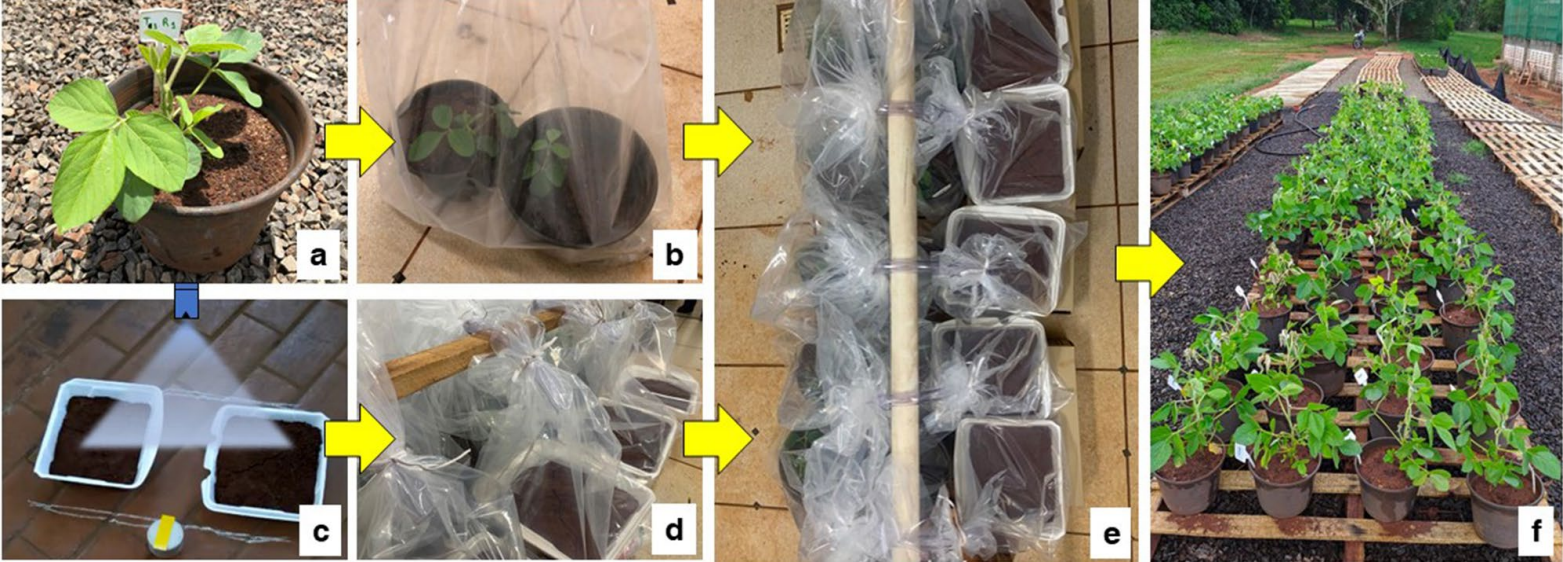
Proposed methodology stages for dicamba volatility evaluation: soybean plants were placed in plastic bags (**a**,**b**); trays containing soil were sprayed with each dicamba tank-mixture (**c**); soil-containing trays were placed inside plastic bags immediately after application (**d**); treated soil and soybean plant bags were connected with atoxic hose and bags were hermetically sealed (**e**); 36 h after application, soybean plants were placed outside (**f**).

Immediately after spraying, each tray containing treated soil was placed inside a polypropylene plastic bag (0.40 × 0.60 × 0.017 m) and sealed with a non-toxic hose outlet of 0.0953 m diameter and 0.35 m length. Hoses were used to allow gas exchange between the plastic bag containing treated soil and the other plastic bag containing two pots with soybean plants. This system composed of two plastic bags containing treated soil and soybean plants was hermetically sealed for 36 h in controlled conditions (27.5 °C and 55% RH). After 36 h, soybean plants pots were removed from plastic bags and kept outside until final phytotoxic evaluations. Methodology phases are illustrated in Fig. 1.

Visual phytotoxicity assessments were performed at 7, 14, 21, and 28 days after application using non-treated controls as comparative standards. Volatility injury ratings of 100% represented dead plants and 0% ratings indicated no herbicide symptoms. Characteristic dicamba symptoms to assess volatility injury included trifoliate folding edges, leaf cupping and stippled leaves, particularly in younger leaves^21^.

Soybean plants were clipped at soil level on January 20, 2020. Each harvested plant was individually placed in paper bags, dried in oven at 60 °C for 48 h. After drying, treatments were weighed on precision scale to determine dry mass weight per soybean plant. Individual yield values of potted soybean plants were evaluated by harvesting all grains of each plant by treatment, followed by weighing the total mass of grains produced per plant on a precision scale. After evaluating individual yield of each plant, 50 grains were randomly selected from each plant repetition and were measured using a precision scale.

### Statistical analysis

The normality and homogeneity of variances were verified by Shapiro–Wilk and Bartlett tests at 5%, respectively. Normal and homogeneous data was submitted to an analysis of variance (ANOVA), and mean values were compared by Scott–Knott test (*p* < 0.05) using the ExpDes.pt package in R software version 4.0.0^22,23^. The results of phytotoxicity, dry mass, pot yield, and 50 grain mass of soybean plants were analyzed using ANOVA by Tukey's test (*p* < 0.05) with SAS software v.9.4 (SAS Institute, Cary, NC, USA)^24^.

## Results and discussion

### Electrical conductivity and pH

**Figure 2:**
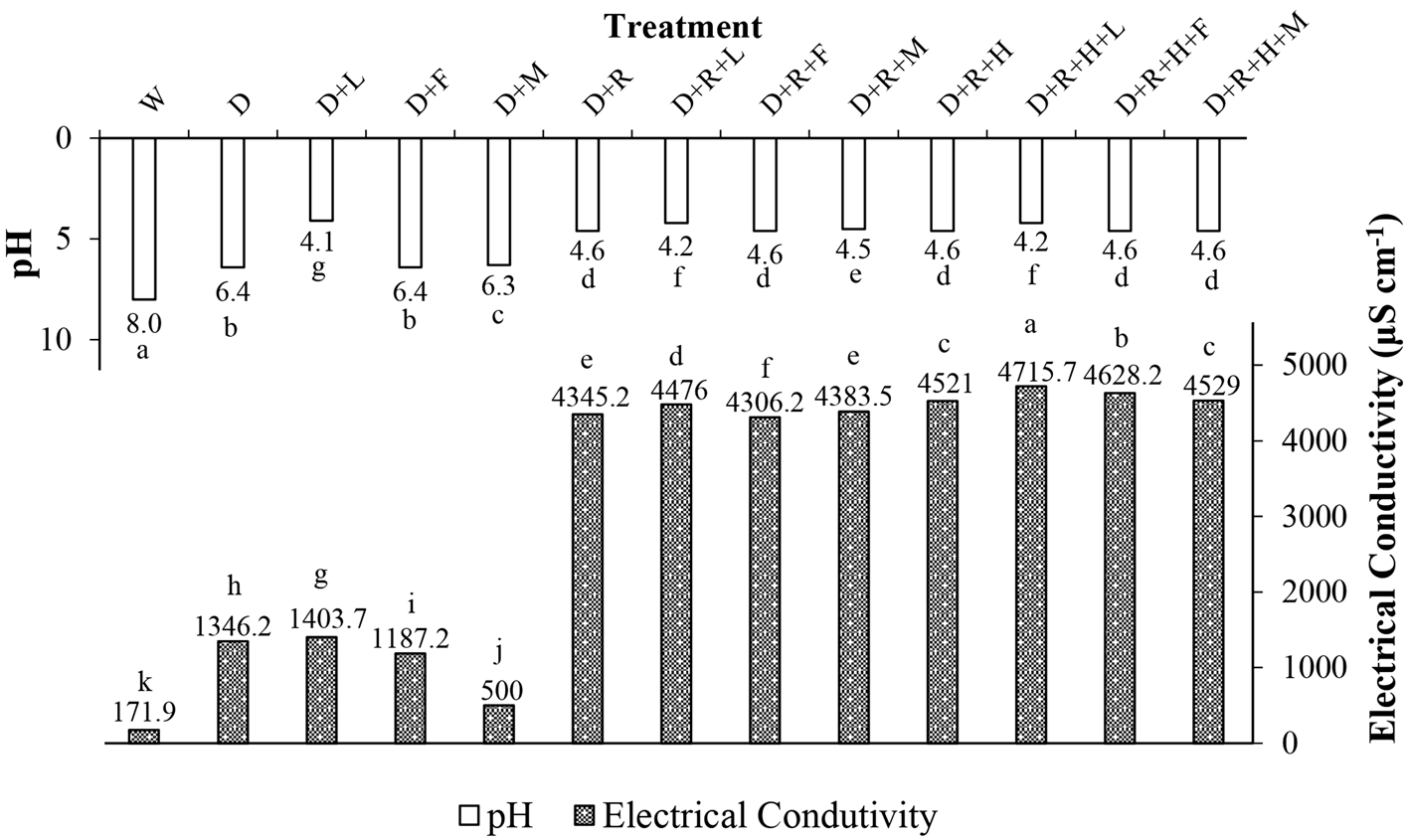
Potential of hydrogen (pH) values and electrical conductivity (μS cm^−1^) of treatments. Abbreviations were as follows: W = Water; D = Dicamba; L = LI700; F = Fluilflex; M = MEES; R = Roundup Transorb R; H = Heat. Bars with mean values followed by same letter within each parameter (pH and electrical conductivity) are not different at α = 0.05.

Results of pH and electrical conductivity were significantly different (*p* < 0.0001) and varied depending on herbicide and adjuvant tank-mixture combination. The pure water treatment (W) presented higher pH value (8.0) compared to other treatments (Fig. 2). The addition of lecithin + methyl ester of soybean + ethoxylated alcohol adjuvant (F) to tank-mixtures containing herbicides did not change pH while the lectin and propionic acid adjuvant (L) reduced pH for all herbicide combinations (Fig. 2). Moreover, glyphosate (R) considerably reduced pH of all tank-mixtures, except for those with lectin and propionic acid adjuvant which already had low pH without glyphosate. In general, tank-mixtures with pH values ranging from 3.5 to 5.5 may favor herbicide activity^25^ due to the reduction of herbicide alkaline hydrolysis. Therefore, as lectin and propionic acid (L) adjuvant acted as an acidifier, it may possibly enhance herbicide control efficacy. On the other hand, the auxinic dicamba herbicide is a weak acid and its molecular state may strongly impact its volatility^26^. Studies have shown a decrease in dicamba volatility when increasing the tank-mixture pH which helped reduce contamination risk of surrounding areas^26^. Researchers have also reported pH decrease when adding glyphosate to spray solutions as observed in the present study^9^. It has been suggested that dicamba formulations with pH modifiers, when tank-mixed with glyphosate, may potentially increase herbicide volatilization^9^.

The lowest values of electrical conductivity were found for water (W) and dicamba mixed with methylated soybean oil (D + M) (171.9 and 500.0 μS cm^−1^, respectively). Cream formation was observed for the mixture of dicamba and methylated soybean oil (D + M) which may have interfered in electrical conductivity results by reducing the concentration of free ions in the spray solution. The dicamba only treatment (D); dicamba mixed with lectin and propionic acid (D + L) and dicamba mixed with lecithin + soybean methylated ester + ethoxylated alcohol (D + F) adjuvants presented intermediate electrical conductivities values (1346.2, 1403.7 and 1187.2, respectively) (Fig. 2).

All treatments containing glyphosate and saflufenacil had high conductivity values with no considerable differences across them. Although high electrical conductivities are associated with low pH values (Fig. 2), elevated electrical conductivity values may favor herbicide absorption and translocation through large ion availability^27^.

Thus, results of volatilization risks related to pH reduction and ion availability showed treatments with dicamba only (D) and with dicamba and lecithin + methyl ester of soybean + ethoxylated alcohol (D + F) presented better values across treatments.

### Surface tension and contact angle

**Figure 3:**
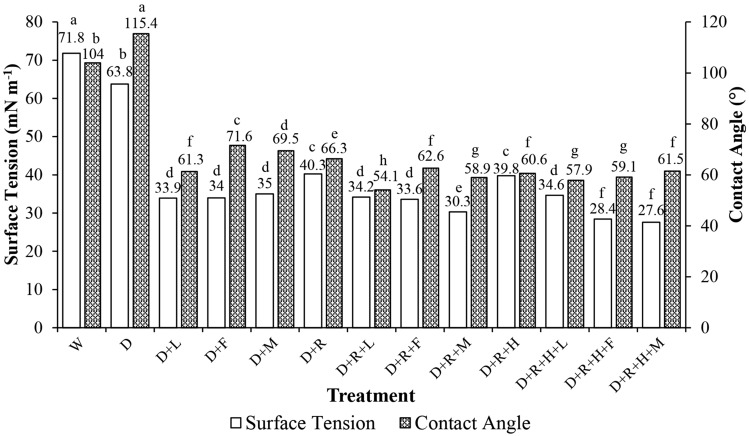
Surface tension and contact angle results (*p* value < 0.0001) of each tank-mixture tested. Abbreviations were as follows: UT = Untreated; D = Dicamba; L = LI700; F = Fluilflex; M = MESS; R = Roundup Transorb R; H = Heat. Bars with mean values followed by same letter within each bar parameter (surface tension and contact angle) are not different at α = 0.05.

Significant differences were observed for contact angle and surface tension results (*p* < 0.0001) as function of herbicide and adjuvant addition to dicamba tank-mixture (Fig. 3). Therefore, herbicide and adjuvant addition affected physicochemical characteristics of spray solutions.

The dicamba only treatment (D) had one of the highest contact angle and surface tension values (115.4° and 63.8 mN m^−1^, respectively) (Fig. 3). Water, the main constituent in spraying solutions, is characterized with high surface tension which, in most cases, will reduce the spread of droplet on plant surfaces after droplet deposit. This characteristic directly influences pesticides efficacy as droplet coverage and droplet size will affect droplet evaporation and absorption rate in plants^28,29^. As also observed in the present study, adjuvants may reduce surface tension and contact angle values when compared to herbicide mixtures without adjuvants^30^.

Therefore, tank-mixtures containing only herbicides without any adjuvants (D, D + R and D + R + H) presented the highest values of surface tension (63.8, 40.3 and 39.8 mN m^−1^, respectively). Because of natural high surface tension of some pesticides, for instance, surfactant adjuvants are commonly added to initial pesticide formulations aiming to reduce the surface tension^31^.

The lowest surface tension values among treatments were obtained with dicamba + potassium glyphosate + saflufenacil + methylated soybean oil (D + R + H + M) and with dicamba + potassium glyphosate + saflufenacil + lecithin + methylated soybean ester + ethoxylated alcohol (D + R + H + F) (27.6 and 28.4 respectively). The lowest contact angle value was observed in the dicamba + potassium glyphosate + lecithin + propionic acid (D + R + L) mixture (54.1°). Some authors have also demonstrated the efficacy of organosilicon adjuvants in reducing the surface tension of spray solutions^31–33^. Treatments with dicamba mixed with potassium glyphosate (D + R) and mixed with potassium glyphosate + saflufenacil (D + R + H) showed contact angle values similar to all treatments except water (W) and dicamba only (D). The surface tension values of D + R (40.3 mN m^−1^) and D + R + H (39.8 mN m^−1^) were considerably higher than the other treatments being only lower than water and dicamba only 71.8 and 63.8 mN m^−1^ respectively).

Although mixtures with adjuvants presented differences in surface tension and contact angle results, these differences were relatively small and followed a pattern within treatments. Such pattern indicated an interaction between dicamba and other active ingredients. In general, low values of surface tension and contact angle indicates greater droplet spread on leaf surface leading to greater spray coverage^31,33^. Surface tension and contact angle information of different tank-mixtures are important and must be considered when spraying herbicides as they affect droplet deposit and coverage which influences herbicide mobility through leaf surface and weed control.

Although treatments with lower surface tension and contact angle values indicated greater droplet spreading over surface, these same treatments (D + R + H + M and D + R + L) presented low pH values (Fig. 2) which increases potential for dicamba volatilization^9^. Considering surface tension, contact angle but also pH, the treatment composed of dicamba + lecithin + soybean methylated ester + ethoxylated alcohol (D + F) had high pH and satisfactory values of surface tension and contact angle.

### Droplet size

Evaluating droplet size results according to the ANSI/ASABE^16^ classification methodology, the MUG11003 nozzle produced the largest droplet size (D_V0.5_) with an Ultra-Coarse droplet classification (Table 4). Both the TTI11003 and ULDM13003 nozzles produced Extremely-Coarse droplets as in the ANSI/ASABE classification. The MUG nozzle had the lowest volume of droplets smaller than 150 µm (%V < 150 µm), 3.3%, and the best droplet spectra uniformity (RS), 1.50, among tested nozzles (Table 4). Considering nozzle drift potential reduction, the MUG nozzle had better results across nozzle types. The ULDM presented the second-best drift potential reduction.

**Table 4:** ANSI/ASABE S572.1 reference nozzles used for droplet size classification and air-induction nozzle results for the following tested parameters: D_V0.1_, D_V0.5_, D_V0.9_, % droplets smaller than 150 µm and relative span (RS).

Nozzle	Pressure	D_V0.1_	D_V0.5_	D_V0.9_	% < 150 μm	RS	ANSI/ASABE S572.3 classification
	kPa	μm					
11001	450	50.9	112.7	209.1	71.9	1.39	VF/F
11003	300	78.3	200.3	397.0	34.3	1.58	F/M
11006	200	111.0	284.1	571.0	18.9	1.62	M/C
8008	250	172.1	382.8	646.7	7.3	1.43	C/VC
6510	200	140.7	446.0	947.0	11.3	1.80	VC/XC
ULDM 13003	250	264.4	713.6	1349.9	3.9	1.52	XC
TTI 11003	250	223.5	732.3	1473.2	4.6	1.70	XC
6515	150	251.2	763.8	1567.0	5.1	1.71	XC/UC
MUG 11003	250	394.65	1072.0	2000.0	3.3	1.50	UC

Abbreviations were as follows: VF, Very Fine; F, Fine; M, Medium; C, Coarse; VC, Very Coarse; XC, Extremely Coarse, UC, Ultra Coarse.

Dicamba treatments and air induction nozzle type affected droplet size, volume of droplets smaller than 150 µm, and RS. The nozzle type with the largest droplet size (DV_0.5_) across treatments was the MUG11003 (Table 5). Similar results were observed when comparing the same MUG, TTI, and ULDM nozzles sprayed with the same tank-mixture (D + R + H + M)^34^. In the same study, higher D_V0.5_ was also produced by the MUG nozzle, 1022 µm, at 300 kPa pressure^34^. Treatments with water (W) and dicamba only (D) produced the largest droplet size for all nozzles and for studied parameters (D_V0.1_, D_V0.5,_ D_V0.9,_ %V < 150 µm, and RS) (Table 5).

**Table 5:** Droplet size results in μm (D_V0.1_, D_V0.5_, D_V0.9_), volume percentage of droplets smaller than 150 µm and relative span (RS) of all tested nozzles and tank-mixtures at constant 250 kPa pressure.

	D_V0.1_			D_V0.5_			D_V0.9_			% < 150 μm			RS		
Treatments	MUG	TTI	ULDM	MUG	TTI	ULDM	MUG	TTI	ULDM	MUG	TTI	ULDM	MUG	TTI	ULDM
W	546^Aa^	333^Ba^	321^Bb^	1212^Aa^	891^Ba^	912^Bb^	2308^Aa^	1795^Ca^	2094^Ba^	1.33^Bb^	2.39^Ad^	3.00^Ac^	1.45^Ab^	1.64^Bb^	1.96^Aa^
D	554^Aa^	327^Ca^	383^Ba^	1291^Aa^	900^Ca^	1100^Ba^	2400^Aa^	1798^Ca^	2199^Ba^	1.27^Bb^	2.62^Ad^	2.39^Ac^	1.43^Bb^	1.63^Ab^	1.66^Ab^
D + L	359^Ac^	217^Bc^	242^Bc^	858^Ab^	618^Bd^	638^Bd^	1793^Ab^	1502^Bb^	1164^Cc^	2.32^Ca^	4.97^Aa^	4.15^Bb^	1.66^Ba^	2.08^Aa^	1.44^Cb^
D + F	396^Ab^	235^Bc^	238^Bc^	850^Ab^	614^Bd^	686^Bd^	1475^Ac^	1107^Bc^	1362^Ab^	1.85^Bb^	4.27^Ab^	3.95^Ab^	1.27^Bc^	1.42^Bc^	1.61^Ab^
D + M	434^Ab^	223^Bc^	238^Bc^	876^Ab^	633^Bd^	695^Bd^	1422^Ac^	1199^Ac^	1295^Ac^	2.48^Ba^	5.56^Aa^	5.53^Aa^	1.13^Bd^	1.54^Ab^	1.51^Ab^
D + R	347^Ac^	299^Ab^	309^Ab^	931^Ab^	784^Bb^	863^Ab^	1653^Ab^	1408^Bb^	1546^Ab^	2.55^Aa^	2.70^Ad^	2.99^Ac^	1.40^Ab^	1.41^Ac^	1.44^Ab^
D + R + L	413^Ab^	272^Bb^	277^Bb^	877^Ab^	693^Bc^	710^Bd^	1445^Ac^	1240^Ac^	1268^Ac^	1.75^Bb^	3.54^Ac^	3.99^Ab^	1.17^Bd^	1.40^Ac^	1.40^Ab^
D + R + F	386^Ab^	281^Bb^	281^Bb^	858^Ab^	701^Bc^	778^Bc^	1484^Ac^	1271^Bc^	1461^Ab^	1.78^Bb^	2.83^Ad^	2.87^Ac^	1.28^Bc^	1.41^Ac^	1.51^Ab^
D + R + M	443^Ab^	277^Bb^	234^Bc^	853^Ab^	685^Bc^	640^Bd^	1353^Ac^	1212^Ac^	1186^Ac^	1.02^Cb^	3.02^Bc^	4.00^Ab^	1.06^Bd^	1.36^Ac^	1.49^Ab^
D + R + H	306^Ad^	262^Bc^	222^Bc^	895^Ab^	711^Bc^	689^Bd^	1707^Ab^	1334^Bb^	1515^Bb^	3.17^Ba^	3.29^Bc^	4.39^Ab^	1.56^Ba^	1.50^Bb^	1.87^Aa^
D + R + H + L	436^Ab^	286^Bb^	288^Bb^	878^Ab^	712^Bc^	749^Bc^	1638^Ab^	1279^Bc^	1399^Bb^	1.39^Bb^	2.79^Ad^	2.89^Ac^	1.26^Bc^	1.39^Ac^	1.47^Ab^
D + R + H + F	428^Ab^	289^Bb^	300^Bb^	889^Ab^	695^Bc^	769^Bc^	1505^Ac^	1334^Ab^	1464^Ab^	1.24^Bb^	2.01^Ad^	2.55^Ac^	1.21^Bc^	1.51^Ab^	1.50^Ab^
D + R + H + M	427^Ab^	232^Cc^	285^Bb^	843^Ab^	576^Bd^	788^Ac^	1366^Ac^	1119^Bc^	1499^Ab^	1.54^Bb^	3.84^Ab^	3.52^Ac^	1.11^Bd^	1.54^Bb^	1.53^Bb^
*p*-value	0.0000			0.0035			0.0000			0.0000			0.0000		

Abbreviations were as follows: W = Water; D = Dicamba; L = LI700; F = Fluilflex; M = MEES; R = Roundup Transorb R; H = Heat. Means followed by same lowercase letter within each column (D_V0.1_, D_V0.5_, D_V0.9_, % < 150 μm, RS) and mean values followed by same uppercase letter within each line (treatments) are not different at α = 0.05.

The lowest values of D_V0.1_ were produced by the TTI and ULDM nozzles, whereas the highest D_V0.1_ value was achieved by the MUG nozzle in all treatments (Table 5). Some treatments had greater effect on droplet size than others. The dicamba + potassium glyphosate + saflufenacil + methylated soybean oil (D + R + H + M) treatment, for example, had the lowest D_V0.5_ across treatments when sprayed with the MUG11003 (843 µm) and TTI11003 (576 µm). The dicamba + lecithin + propionic acid (D + L) treatment sprayed with ULDM13003 was the one that most reduced its D_V0.5_ value, from 1100 µm with dicamba only (D) to 638 µm with adjuvant (D + L) addition. It was observed less D_V0.5_ variation for treatments sprayed with TTI nozzle, with standard deviation of 104 µm, while ULDM and MUG nozzles had greater variations, with standard deviations of 154 and 153 µm, respectively.

Any adjuvant and herbicide added to dicamba tank-mixture promoted lower droplet sizes for nozzles. Similar results were observed when dicamba was tank-mixed with *S*-metolachlor^35^. The MUG nozzle with the dicamba only, for example, produced droplets (D_V0.5_) of 1291 µm. When any adjuvant was added to tank-mixture, such as LI700 (D + L), the D_V0.5_ dropped to 858 µm, a reduction of 33.5%. There was a reduction of droplet size with the TTI nozzle of up to 36% when comparing droplet size results of dicamba only (D) and dicamba + potassium glyphosate + saflufenacil + methylated soybean oil (D + R + H + M). Up to 42% droplet size reduction was observed when using the ULDM nozzle comparing dicamba only (D) and dicamba + lecithin + propionic acid (D + L) tank-mixtures.

Regarding driftable droplet percentage (%V < 150 µm), the MUG nozzle presented the lowest percentage among nozzles and treatments (Table 5). TTI and ULDM both had similar driftable droplet percentage results, with result variation ranging from 2 to 5.5%. These results are similar to results observed by other authors also studying droplet size of dicamba with air-induction nozzles^34^. Regarding drift risk, dicamba + potassium glyphosate + saflufenacil (D + R + H) tank-mixture was the treatment that most increased the risk of drift. Considering the MUG nozzle results, for example, dicamba only (D) results of driftable droplet volume where 1.27%. With the D + R + H treatments, its value increased to 3.17%. Droplet spectra uniformity (RS) was better obtained with the MUG nozzle, an average of 1.31 RS value; followed by the TTI nozzle, 1.52 of RS; followed by the ULDM nozzle, 1.57 of RS.

Considering only droplet size results objecting drift reduction, the dicamba only treatment (D) was the most effective across treatments. However, when also considering surface tension and contact angle results (Fig. 3), the dicamba only treatment (D) has low droplet spread potential which may negatively affect herbicide efficacy. Adding an adjuvant to dicamba tank-mixture that does not reduce its pH, droplet size while it increases its droplet spread would be the best scenario, considering herbicide efficacy and volatility. Some non-ionic surfactant adjuvants, for example, can reduce tank-mixtures surface tension and contact angle while it can increase droplet size^36^. Future studies should include non-ionic surfactant adjuvants to determine its effects on physicochemical characteristics, droplet size and volatility of dicamba tank-mixtures.

### Dicamba volatility

**Figure 4:**
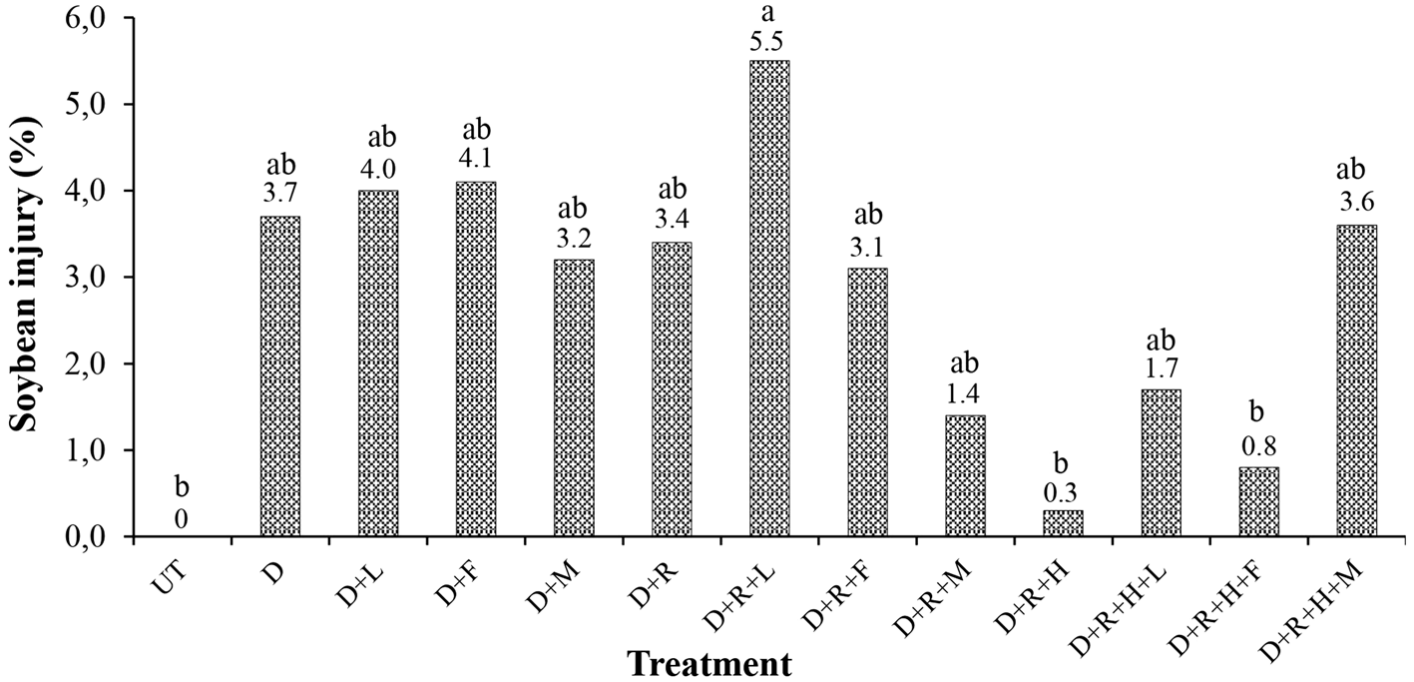
Soybean injury results of dicamba exposed plants at 14 days after application (DAA) of each treatment. Abbreviations were as follows: UT = Untreated; D = Dicamba; L = LI700; F = Fluilflex; M = MESS; R = Roundup Transorb R; H = Heat. Bars with mean values followed by same letter are not different at α = 0.05.

There were no significant differences between phytotoxicity treatment results at 7 DAA (*p* = 0.6438), 21 DAA (*p* = 0.6388), and 28 DAA (*p* = 0.6530) (Table 6). At 14 DAA, however, significant differences were observed (*p* = 0.0002) (Fig. 4). The dicamba + potassium glyphosate + lecithin + propionic acid (D + R + L) treatment presented the highest phytotoxicity rating (5.5%) at 14 DAA (Fig. 4). The same treatment (D + R + L) also presented the highest numerical phytotoxicity rating values at 21 and 28 DAA (3.4 and 5.5%, respectively) as in Table 6. Greater dicamba injury was also observed at 14 DAA in a simulated dicamba drift study^37^.

**Table 6:** Soybean injury results of dicamba exposed plants at 7, 21 and 28 days after application (DAA) of each treatment.

Treatment	Soybean injury (%)		
	7 DAA	21 DAA	28 DAA
UT	0.0 ns	0.0 ns	0.0 ns
D	0.8	2.5	4.4
D + L	0.3	1.7	2.3
D + F	0.3	1.4	2.5
D + M	0.3	2.8	2.8
D + R	0.3	2.2	2.8
D + R + L	0.6	3.4	5.5
D + R + F	1.1	0.0	0.6
D + R + M	0.6	0.6	2.8
D + R + H	0.0	0.6	1.9
D + R + H + L	0.4	0.2	1.9
D + R + H + F	0.0	0.0	0.8
D + R + H + M	0.7	1.2	3.1
*p* value	0.6438	0.6388	0.6530

Abbreviations were as follows: UT = Untreated; D = Dicamba; L = LI700; F = Fluilflex; M = MESS; R = Roundup Transorb R; H = Heat. Means were not significant at 7, 21 and 28 DAA at α = 0.05.

**Figure 5:**
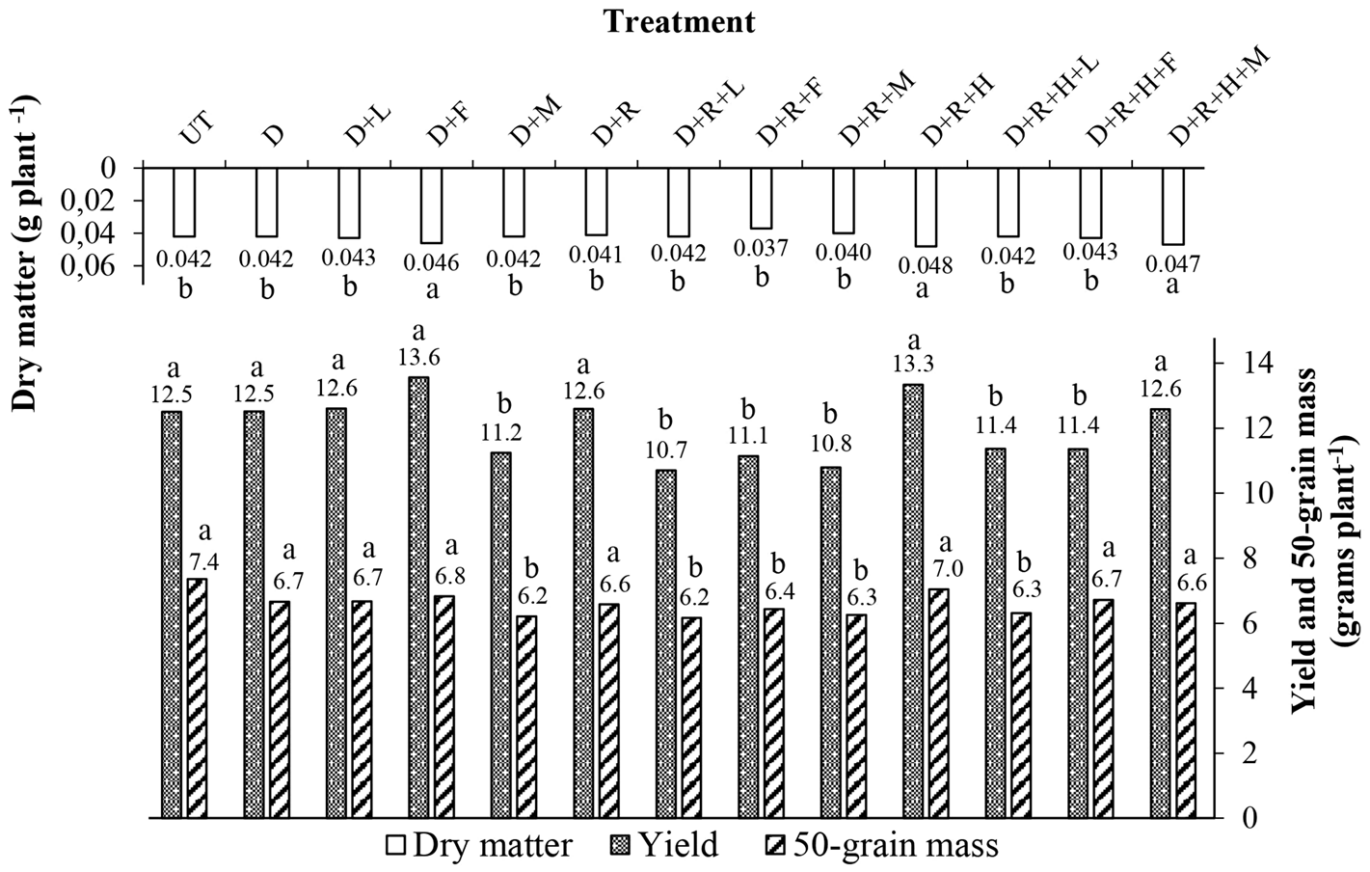
Soybean yield (g plant^−1^), 50-grain mass weight (g plant^−1^) and dry matter weight (g plant^−1^) of each treatment. Abbreviations were as follows: UT = Untreated; D = Dicamba; L = LI700; F = Fluilflex; M = MESS; R = Roundup Transorb R; H = Heat. Bars with mean values followed by same letter within each parameter (yield, 50-grain mass, dry matter) are not different at α = 0.05.

Soybean yield was significantly affected by dicamba exposure (*p* = 0.0013). The highest yield results in soybean pots were obtained on plants exposed by dicamba + lecithin + methyl ester of soybean + ethoxylated alcohol (D + F) and by dicamba + potassium glyphosate + saflufenacil (D + R + H) (13.56 and 13.34 g plant^−1^, respectively) (Fig. 5). Lower yield was observed for plants exposed by dicamba + potassium glyphosate + lecithin + propionic acid (D + R + L) (10.70 g plant^−1^) as in Fig. 5.

50-grains mass of soybean plants were significantly affected by dicamba exposure (*p* = 0.0138). The 50-grain mass results indicated higher values for the untreated check (T) and dicamba + potassium glyphosate + saflufenacil (D + R + H) treatment (7.36 and 7.04 g plant^−1^, respectively) (Fig. 5). The lowest 50-grain mass result was obtained for soybean plants exposed by dicamba + potassium glyphosate + lecithin + propionic acid (D + R + L) (6.16 g plant^−1^) as in Fig. 5.

Soybean exposure to dicamba also significantly affected plant dry matter weight (*p* = 0.0130). High dry matter weight results were obtained for plants exposed by dicamba + lecithin + methylated soybean ester + ethoxylated alcohol (D + F), dicamba + potassium glyphosate + saflufenacil (D + R + H), and dicamba + potassium glyphosate + saflufenacil + methylated soybean oil (D + R + H + M) (0.046, 0.048 and 0.047 g plant^−1^, respectively) as in Fig. 5.

Evaluating the results of phytotoxicity (Table 6 and Fig. 4), soybean yield in pots, and 50-grain mass (Fig. 5), it is possible to observe a strong trend for plants exposed by the tank-mixture dicamba + potassium glyphosate + lecithin + propionic acid (D + R + L). This tank-mixture presented the highest phytotoxic rating values at 14, 21, and 28 DAA, the lowest soybean yield and lowest 50-grain mass value. Meanwhile, the same tank-mixture (D + R + L) produced the lowest contact angle, 34.1° (Fig. 1), low surface tension, 34.2 mN m^−1^ (Fig. 3), and presented the second lowest pH value, 4.2 (Fig. 2). Based on the pH and contact angle results, it is hypothesized the high acidity combined with low droplet contact angle and low surface tension within treated soil surface may have enhanced tank-mixture instability increasing dicamba volatilization. It has been proven, for example, that the surface tension of a liquid directly affects its vapor pressure^38,39^. In general, low surface tension values may enhance the vapor pressure of a liquid increasing its volatility. In addition to the surface tension effect, low pH values of dicamba tank-mixtures may also enhance dicamba volatilization^9,26^.

**Figure 6:**
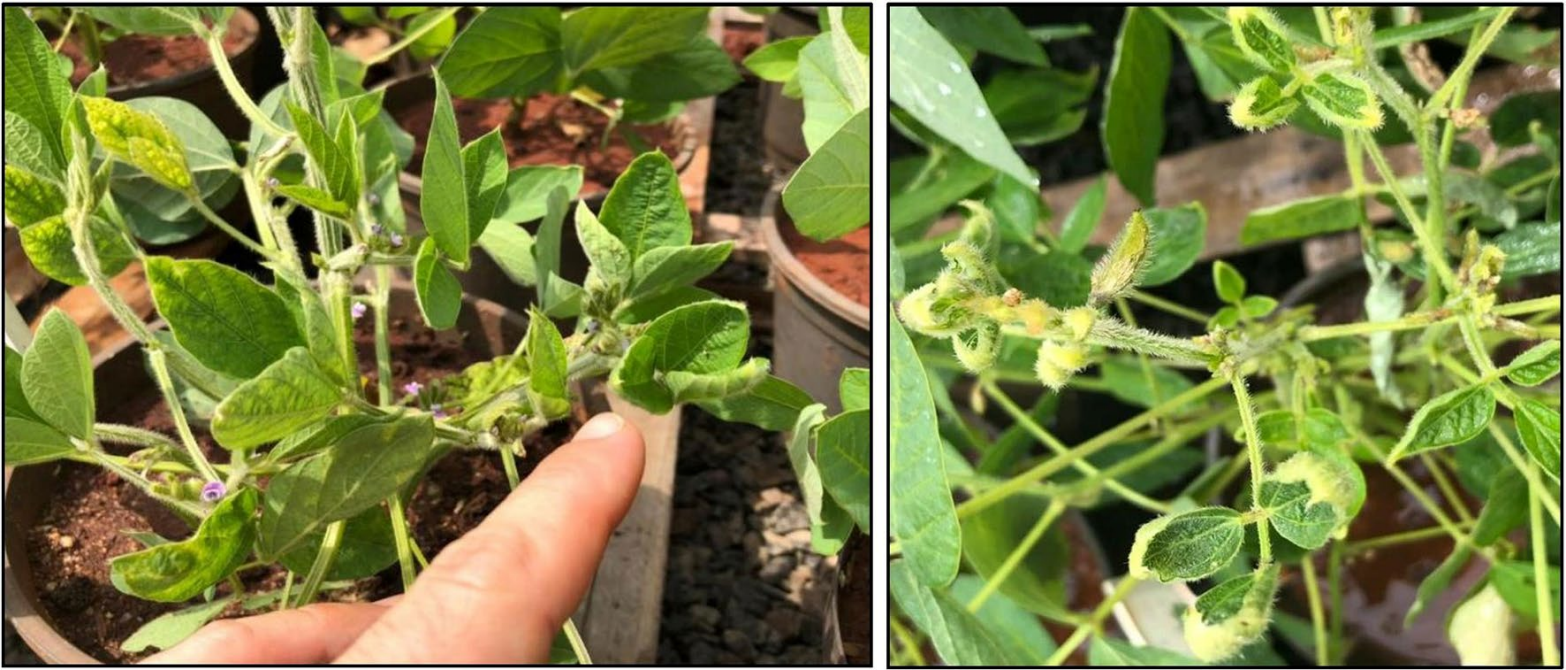
Dicamba injury symptoms observed in exposed soybean plants.

In both volatility replica studies; however, low phytotoxicity results were generally observed. Few soybean plants showed evident and classical dicamba phytotoxicity symptoms (Fig. 6). It is possible that volatilization of dicamba alone and dicamba tank-mixtures was reduced as consequence of high relative humidity inside both bags containing soybean plants and containing treated soil. It is known that high relative humidity can significantly reduce dicamba volatility^40^. It has been observed, for example, that inside hermetically sealed environments, relative humidity can rapidly increase, reaching up to 90%, and may affect the gas exchange dynamics and respiration in plants^41^. Furthermore, it has been shown that concentration of dicamba suspended in air decreases as relative humidity raises from 20 to 50% during 48 h after application^18^.

Another factor that may have influenced phytotoxicity results was the separation of applied soil and soybean plants in different bags, connected in between 9.53 mm diameter hose. This diameter restriction of the hose may have reduced the space for gas exchange between soybean plants and treated soil. Future research with similar methodology should consider placing dicamba treated soil trays and soybean plants in pots in one common sealed environment.

## Conclusions

The tank-mixture of dicamba with lecithin + methyl soybean ester + ethoxylated alcohol adjuvant (D + F) presented the best physicochemical characteristics considering high pH and satisfactory values of conductivity, contact angle, and surface tension.

Drift reduction was better obtained when using the MUG11003 nozzle, producing less amount of driftable droplets and greater droplet size among the tested air induction nozzles.

Dicamba tank-mixed with potassium glyphosate and lecithin + propionic acid (D + R + L) was the most volatile and toxic tank-mixture to exposed soybean plants. Dicamba tank-mixtures with lecithin + methyl soybean ester + ethoxylated alcohol (D + F) and with potassium glyphosate + saflufenacil (D + R + H) showed low injury levels to exposed soybean plants. Despite few visible symptoms of dicamba volatility injury, the proposed method is feasible to evaluate volatilization of dicamba tank-mixtures.

## Data Availability

The dicamba herbicide included in this study was used under license, and so information on this reagent is not publicly available. This information however is available from the authors upon reasonable request such as datasets generated and/or analyzed during the current study.
