# Author Correction: Physicochemical properties, droplet size and volatility of dicamba with herbicides and adjuvants on tank-mixture

**DOI:** 10.1038/s41598-021-84555-5

**Published:** 2021-03-05

**Authors:** Pedro Henrique Urach Ferreira, Leonardo Vinicius Thiesen, Gabriela Pelegrini, Maria Fernanda Tavares Ramos, Matheus Moreira Dantas Pinto, Marcelo da Costa Ferreira

**Affiliations:** Departamento de Ciências da Produção Agrícola, UNESP, Jaboticabal Campus, Jaboticabal, SP Brazil

Correction to: *Scientific Reports* https://doi.org/10.1038/s41598-020-75996-5, published online 02 November 2020

The original version of this Article contained an error.

The product name for the dicamba herbicide was included, however, due to issues relating to confidentiality, it has since been removed from the figures, the text, and tables. This information is available from the authors, and the data availability section has been updated to reflect this.

These errors have now been corrected in the PDF and HTML versions of the Article.

